# Analysis of subgingival micro-organisms based on multi-omics and Treg/Th17 balance in type 2 diabetes with/without periodontitis

**DOI:** 10.3389/fmicb.2022.939608

**Published:** 2022-11-28

**Authors:** Lanlan Jiang, Jiaming Zhang, Meifei Fang, Yingfen Qin, Yuxiao Huang, Renchuan Tao

**Affiliations:** ^1^Department of Periodontics and Oral Medicine, College of Stomatology, Guangxi Medical University, Nanning, China; ^2^Guangxi Health Commission Key Laboratory of Prevention and Treatment for Oral Infectious Diseases, Nanning, China; ^3^Guangxi Key Laboratory of the Rehabilitation and Reconstruction for Oral and Maxillofacial Research, Nanning, China; ^4^Department of Endocrinology, The First Affiliated Hospital, Guangxi Medical University, Nanning, China

**Keywords:** type 2 diabetes, periodontitis, subgingival micro-organisms, metabonomics, T lymphocyte

## Abstract

Type 2 diabetes mellitus (T2DM) and periodontitis are common and interrelated diseases, resulting in altered host response microbiota. The subgingival micro-organisms play a key role in periodontitis pathogenesis. To assess the shift of subgingival microbiome and metabolome in T2DM, we performed an analysis of the subgingival microbiome in patients with T2DM (*n* = 20) compared with non-diabetes (ND) subjects (*n* = 21). Furthermore, patients were subdivided into 10 T2DM with periodontitis (DP), 10 T2DM without periodontitis (DNP), 10 periodontitis (P), and 11 healthy control (H) groups. 16SrRNA gene sequencing combined with ultra high-performance liquid chromatography-mass spectrometry (UHPLC–MS) based metabolomics was performed in all participants. T lymphocyte immunity was analyzed by flow cytometry. Furthermore, the network relationship among subgingival micro-organisms, metabolites, blood glucose level, and T lymphocyte immunity were analyzed. The results showed that the difference of the subgingival microbiome from healthy to periodontitis status was less prominent in T2DM compared with ND, though the clinical signs of disease were similar. The bacteria *Eubacterium nodatum* group, *Filifactor*, *Fretibacterium*, *Peptostreptococcus*, and *Desulfovibrio*, amongst others, may be important in the pathopoiesia of periodontitis in the T2DM state. In addition, some dominant bacteria showed network relationships. The Treg/Th17 ratio was lower in the DP and DNP groups than in the P and H groups—though that of P was lower than for H. The percentage of CD4^+^/CD8^+^ PD1 and CD8^+^ PDL1 was higher in the DP and DNP groups than in the H group; the percentage of CD8^+^ PDL1 was higher in the DP than P groups. Subgingival micro-organisms in periodontitis had a significant metabolic shift in terms of their signature metabolites. Butyrate metabolism and phenylalanine metabolism may play a role in the pathogenesis of periodontitis with/without T2DM. Specifically, biphenyl degradation, tryptophan metabolism, and the two-component system may play important roles in periodontitis with T2DM. Lastly, the network relationship among subgingival micro-organisms, metabolites, blood glucose level, and T lymphocyte immunity were unbalanced. This study identified the changes in the subgingival microbiome associated with periodontitis in T2DM, as well as the associated network between bacterial flora, metabolism dysbiosis, and immune regulation.

## Introduction

Diabetes mellitus is a systemic disease at globally epidemic proportions, and the number of individuals with type 2 diabetes mellitus (T2DM) has dramatically increased worldwide in the past few decades (Hu and Jia, 2018). It has been suggested by clinical evidence that T2DM increases the risk of developing inflammatory diseases such as periodontitis—an oral disease affecting more than half of patients with T2DM (Preshaw et al., 2012; Verhulst et al., 2019). Diabetes upregulates inflammatory response and promotes tissue destruction, while periodontal inflammation can affect blood glucose metabolism in patients with T2DM through insulin resistance (Lalla and Papapanou, 2011; Taylor et al., 2013). Although various studies have confirmed a two-way relationship between diabetes and periodontitis (Preshaw et al., 2012), the specific mechanism of their interaction remains unclear.

T2DM patients may be more susceptible to shifts in the subgingival microbiome toward dysbiosis (Longo et al., 2018; Shi et al., 2020). Although there are many studies, there is no consistent conclusion (Shi et al., 2020; Balmasova et al., 2021). Subgingival microbiota metabolites may be an important role player between diabetes and periodontitis. Periodontal microbial antigen components, toxins, metabolites, and enzymes, can altogether contribute to local inflammation, periodontal tissue destruction, and once circulated into deep tissue areas stimulate the host immune response and systemic inflammatory response (Cunningham et al., 2014; Kumar, 2017; Sudhakara et al., 2018). Nevertheless, the metabolic function of the subgingival microbiome and immunity associated with concurrent T2DM and periodontitis have not yet been investigated.

A variety of periodontal pathogens (*Porphyromonas gingivalis*, *Prevotella intermedia,* etc.) have been confirmed to inhibit T lymphocyte cell function (Do Vale et al., 2004; Gaddis et al., 2013). For instance, *P. gingivalis* can up-regulate the expression of PD1/PDL1 in CD4^+^T lymphocytes (Keir et al., 2008). The PD1/PDL1 signaling pathway, comprised of the programmed cell death protein 1 (PD1) and programmed cell death ligand 1 (PDL1) expressed on the surface of T lymphocyte cells, can inhibit the proliferation of T lymphocyte cells and down-regulate specific T lymphocyte immune function to limit immune-mediated host tissue damage and promote the persistence of infection (Francisco et al., 2010; Noack and Miossec, 2014). The PD1/PDL1 pathway is closely related to Treg proliferation, and PDL1 can lead to Treg cell differentiation and maintain its function. In contrast, PDL1 deficiency causes impaired Tregs *in vivo* (Mcgee et al., 2010). Meanwhile, the PD1/PDL1 pathway controls the complex dynamic interactions with effector T cells (Francisco et al., 2010; Gianchecchi et al., 2013). Low levels of inflammation are also likely to cause insulin resistance. Despite progress made in our understanding of subgingival micro-organism interactions, the correlation and network relationship between their metabolites, blood glucose level effect, and T lymphocyte immunity in T2DM with/without periodontitis states is still unknown.

Therefore, this study analyzed the bacterial species of the subgingival microbiome, their metabolome, and their role in T lymphocyte immunity among patients with/without T2DM and/or periodontitis. We furthermore determined the species compositions and the correlative roles of subgingival microbiome metabolites in patients with T2DM and periodontitis. Lastly, we aimed to demonstrate the relationship among species, metabolites, and T lymphocyte immunity associated with periodontitis in T2DM.

## Materials and methods

### Study population, selection criteria, and sample collection

The study was performed at the Guangxi Medical University College of Stomatology and the First Affiliated Hospital of Guangxi Medical University, Nanning Guangxi, China. The study was approved by the Ethical Committee of the Guangxi Medical University (protocol number: #2020010). Each subject provided written informed consent before participation. All clinical procedures were performed in accordance with the Declaration of Helsinki and Good Clinical Practice Guidelines.

The inclusion criteria of participants conform to the World Health Organization or American Diabetes Association criteria and periodontitis 1999 International Classification for periodontal disease, and participants were divided into four groups, including 10 patients with T2DM and periodontitis (DP), 10 patients with T2DM without periodontitis (DNP), 10 patients with periodontitis (P), and 11 healthy controls (H). An initial full-mouth examination for all participants was performed at the first visit to assess the clinical parameters of the periodontium including the gingival retraction, probing depth (PD), attachment level (AL), and bleeding on probing. Tooth sites with PD ≥ 5 mm and bleeding on probing at the first evaluation visit were considered diseased and sampled sites. Individuals with healthy periodontium were defined as presenting with PD ≤4 mm and minimal bleeding on probing (<10–15% of all sites); a tooth site with no bleeding on probing were considered sampled sites. The majority of samples were taken from molar or premolar tooth sites in the T2DM and ND subjects. All subjects were required to abstain from any mouth rinse with antibiotics 2 months prior to sampling, and abstain from localized scaling and root planing for 3 months prior to sampling. For all T2DM subjects, the blood glucose and glycated hemoglobin (HbA1c) levels were recorded. All ND subjects had a blood glucose level < 6.66 mM.

The exclusion criteria were as follows: pregnancy, lactation, use of antibiotic, anti-inflammatory, or immunosuppressive therapies within the last 6 months, history of smoking, long-term use of mouth rinses containing antimicrobials, use of orthodontic appliances, presence of other systemic conditions (e.g., cardiovascular, cerebral diseases, immunological disorders or osteoporosis, etc.).

The subgingival plaques were collected by sterile curettes, suspended directly in frozen storage tubes (containing 1 ml sterile double-steamed water), and immediately stored at −80°C till further analysis.

### 16S rRNA microbial community analysis

DNA from different samples was extracted from tool using the E.Z.N.A. ®Stool DNA Kit (D4015, Omega, Inc., United States) according to manufacturer’s instructions. To analyze the taxonomic composition of the bacterial community, the V3-V4 region of the 16S rRNA gene was selected for the subsequent pyrosequencing. Amplicon polymerase chain reaction (PCR) was performed for the 16S rRNA hypervariable region V3-V4 (Primers: 338F (5’-ACTCCTACGGGAGGCAGCAG-3); 806R (5’-GGACTACHVGGGTWTCTAAT-3′)). The 5′ ends of the primers were tagged with specific barcods per sample and sequencing universal primers. The PCR products were purifyied by AMPure XT beads (Beckman Coulter Genomics, Danvers, MA, United States) and quantified by Qubit (Invitrogen, United States). The amplicon pools were prepared for sequencing and the size and quantity of the amplicon library were assessed on Agilent 2,100 Bioanalyzer (Agilent, United States) and with the Library Quantification Kit for Illumina (Kapa Biosciences, Woburn, MA, United States), respectively. The libraries were sequenced on NovaSeq PE250 platform. Samples were sequenced on an Illumina NovaSeq platform according to the manufacturer’s recommendations, provided by LC-Bio. Paired-end reads was assigned to samples based on their unique barcode and truncated by cutting off the barcode and primer sequence. Paired-end reads were merged using FLASH. Quality filtering on the raw tags were performed under specific filtering conditions to obtain the high-quality clean tags according to the fqtrim (V 0.94). Chimeric sequences were filtered using Vsearch software (v2.3.4). Sequences with ≥97% similarity were assigned to the same operational taxonomic units (OTUs) by Vsearch (v2.3.4). Representative sequences were chosen for each OTU, and taxonomic data were then assigned to each representative sequence using the RDP (Ribosomal Database Project) classifier. OTUs abundance information were normalized using a standard of sequence number corresponding to the sample with the least sequences. Alpha diversity is applied in analyzing complexity of species diversity for a sample through 5 indices, including Chao1, Observed species, Goods coverage, Shannon, Simpson, and all this indices in our samples were calculated with QIIME (Version 1.8.0). Beta diversity analysis was used to evaluate differences of samples in species complexity. Beta diversity were calculated by (PCoA) and cluster analysis by QIIME software. Blast was used for sequence alignment, and the OTU representative sequences were annotated with RDP (ribosome database) and NCBI-16S database for each representative sequence. Other diagrams were implemented using the R package (V3.4.4).

We searched for bacterial biomarkers of T2DM patient with periodontitis using Linear discriminant analysis (LDA) effect size (LEfSe). The cut-off value was the log value>3.0 and Wilcoxon rank-sum test: *p* < 0.05. The biomarker’s taxon at the genus level was subjected to the logistic regression analysis to develop a diagnostic model.

### Metabolic profiling

#### Sample preparation

The collected samples (20 μl) were extracted with 120 μl precooled 50% methanol, incubated at 25°C for 10 min, and stored overnight at − 20°C. After centrifugation at 4,000 g for 20 min, the supernatants were collected and stored at − 80°C till LC–MS analysis. In addition, pooled QC samples were also prepared by combining 10 μl of each extraction mixture. LC–MS analysis.

All chromatographic separations were performed using an ultra-performance liquid chromatography (UPLC) system (Sciex, Framingham, MA, United States). An ACQUITY UPLC T3 column (100 mm × 2.1 mm, 1.8 μm; Waters, Milford, MA, United States) was used for the reversed-phase separation. A high-resolution tandem mass spectrometer TripleTOF5600plus (Sciex) was used to detect metabolites eluted from the column. The Q-TOF was operated in both positive and negative ion modes. During the acquisition, mass accuracy was calibrated every 20 samples. Furthermore, to evaluate the stability of the LC–MS during the whole acquisition, a quality control sample (pool of all samples) was acquired every 10 samples.

The acquired MS data pre-treatments including peak picking, peak grouping, retention time correction, second peak grouping, and annotation of isotopes and adducts were performed using XCMS v3.16.1. LC–MS raw data files were converted into mzXML format and then processed by the XCMS, CAMERA and metaX toolbox implemented with the R software. Each ion was identified by combining retention time (RT) and m/z data. Intensities of each peaks were recorded and a three dimensional matrix containing arbitrarily assigned peak indices (RT-m/z pairs), sample names(observations) and ion intensity information (variables) was generated. The online KEGG, HMDB database was used to annotate the metabolites by matching the exact molecular mass data (m/z) of samples with those from database. If a mass difference between observed and the database value was less than 10 ppm, the metabolite would be annotated and the molecular formula of metabolites would further be identified and validated by the isotopic distribution measurements. We also used a in-house fragment spectrum library of metabolites to validate the metabolite identidification. The intensity of peak data was further preprocessed by metaX. Those features that were detected in less than 50% of QC samples or 80% of biological samples were removed, the remaining peaks with missing values were imputed with the k-nearest neighbor algorithm to further improve the data quality. PCA was performed for outlier detection and batch effects evaluation using the pre-processed dataset. Quality control-based robust LOESS signal correction was fitted to the QC data with respect to the order of injection to minimize signal intensity drift over time. In addition, the relative standard deviations of the metabolic features were calculated across all QC samples, and those >30% were then removed. Student t-tests were conducted to detect differences in metabolite concentrations between 2 phenotype. The *p* value was adjusted for multiple tests using an FDR(Benjamini–Hochberg). Supervised PLS-DA was conducted through metaX to discriminate the different variables between groups. The VIP value was calculated. A VIP cut-off value of 1.0 was used to select important features.

### Percentage of Treg/Th17 and CD4^+^/CD8^+^ PD1/PDL1 analysis

Blood samples were collected in ethylenediaminetetraacetic acid (EDTA) tubes and processed to extract the peripheral blood mononuclear cell (PBMC) fraction using a Ficoll gradient. Cell staining was performed as manufacturer’s requirements. Briefly, cells were incubated with BB515-conjugated anti-human CD279, APC-conjugated anti-human CD274, AF700-conjugated anti-human CD4 monoclonal antibody (mAb), and PerCP-Cy5.5-conjugated anti-human CD25 mAb for surface staining at 4°C for 20 min, to detect the percentage of CD4^+^ PD1/PDL1. For Tregs detection, the cells were fixed and permeabilized at 25°C for 45 min, and then stained with PE-conjugated anti-human FOXP3 mAb in FOXP3/transcription staining buffer.

For Th17 cell and CD8^+^ PD1/PDL1 detection, the PBMCs were resuspended in a complete RPMI1640 medium with a cell activation cocktail for 5 h in a CO_2_ incubator. Stimulated cells were washed twice with staining buffer, a surface staining protocol was performed, and then cells were incubated with BB515-conjugated anti-human CD279, APC-conjugated anti-human CD274, AF700-conjugated anti-human CD3 mAb, and PerCP-Cy5.5-conjugated anti-human CD8 mAb for surface staining at 4°C for 20 min. In addition, cells were stained with PE-conjugated anti-human IL17A mAb and incubated for 45 min at 25°C. BB515-conjugated rat IgG2a was used as the isotype control for CD279 staining. All the conjugated antibodies and reagents were purchased from BD Bioscience (Franklin Lakes, NJ, United States). All fluorescence-activated cell sorting assays were performed using FACS Canto II (BD Bioscience) and the data were analyzed using FlowJo v10 (Tree Star, Ashland, OR, United States).

### Association analysis between microbial taxa, metabolites and clinical parameters

To determine the association between different genus and secondary metabolites in T2D and/or with/without periodontitis, we constructed a correlation analysis among genus with Relative relation value (r) > 0.3 to get top 10 genus, and a correlation analysis among differential metabolites with r > 0.8 to get top 80 metabolites by Spearman’s correlations in R version 3.2.5. At last, Spearson’s correlation between the top 10 differential genus of two groups, and top 80 metabolites, clinical parameters were computed by the R package stats (version 3.2.5). The network graphs were made using Cytoscape (version 3.7.1).

### Statistical analysis

The Kolmogorov–Smirnov test was used to check for normality; the Mann–Whitney U test was used to compare any two data sets that were not normally distributed; otherwise, one-way ANOVA followed by the Student–Newman–Keuls method was used, and the values were presented as mean ± standard error of the mean (SEM); *p*-values were adjusted by the Benjamini–Hochberg method. Both the T test or Wilcoxon rank-sum test combined with the Benjamini-Hochberg method and a two-stage statistical procedure were applied to compare bacteria taxa. Microbial correlation was estimated using SparCC. The significantly distinguished taxa and predicted pathways by PICRUSt were screened by comparison among groups by the T test or Wilcoxon test. Metabolites with >2-fold, VIP ≥ 1, *p* < 0.05 (T test) between two groups were selected to analyze. Spearman’s rank correlation test and Cytoscape v3.9.1 were used to analyze the relationship among variables including bacteria, metabolites, and clinical parameters; the results were further justified by the empirical permutation test. A two-sided *p* < 0.05 was considered significant.

## Results

### Sample and data collection

A total of 41 participants were enrolled in this study according to the inclusion and exclusion criteria. Table 1 shows the demographic characteristics, as well as the fasting blood glucose (FBG) and HbA1c levels of the patients. There were no significant differences among groups in terms of sex or body mass index (BMI). However, the age of patients with diabetes was higher than those without (*p* < 0.05). Besides, the FBG and HbA1c levels of patients with DP were higher than those with DNP (*p* < 0.05). There was no significant difference in PD, AL, and BI between the patients with DP or P (*p* > 0.05; Table 1).

**Table 1:** Demographic and clinical characteristics of subjects.

Group	T2DM with periodontitis (DP) (n = 10)	T2DM without periodontitis (DNP) (n = 10)	Periodontitis (P) (n = 10)	Healthy (H) (n = 11)
Age	63.00 ± 9.99	64.10 ± 6.29	49.9 ± 7.50	44.18 ± 8.14
Gender				
Male	5	5	5	6
Female	5	5	5	5
Nationality				
Han	9	9	9	9
Minority	1	1	1	2
BMI	23.64 ± 2.57	23.63 ± 6.02	22.78 ± 3.01	22.60 ± 3.85
FBG (mM)	8.41 ± 2.96	6.22 ± 1.76	5.22 ± 0.26	5.02 ± 0.29
HbA1c (%)	7.01 ± 1.32	6.69 ± 0.32	<6.50	<6.50
PD (mm)	4.04 ± 0.69	2.42 ± 0.78	4.51 ± 0.42	------
AL (mm)	4.27 ± 0.99	2.51 ± 0.22	4.64 ± 0.61	------
BI	2.80 ± 0.35	0.95 ± 0.16	3.10 ± 0.39	0.82 ± 0.25

Data are presented as mean ± S.E.M. unless otherwise indicated.

BI, bleeding index; PD, probe depth of the sampling site; AL, attachment loss; BMI, body mass index; T2DM, type 2 diabetes mellitus.

The age of patients with T2DM was higher than those without diabetes (*p* < 0.05); The FBG and HbA1c levels were higher in patients with DP than DNP (*p* < 0.05); There was no significant difference in PD, AL, or BI between the DP and P groups (*p* > 0.05)

### Subgingival microbiota changes of patients

#### Changes of the subgingival microbiome in different periodontal states In T2DM and ND subjects

**Figure 1:**
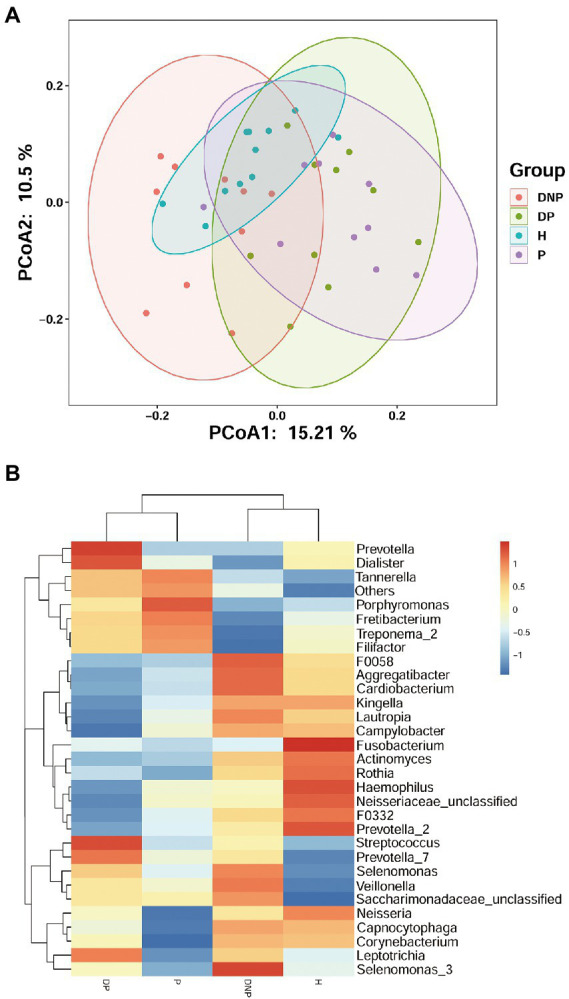
Distribution of differential genera among four groups. **(A)** Principal Co-ordinates Analysis of bacterial communities in different groups. Each symbol represents one sample; Each color represents one group. **(B)** The subgingival microbial compositions profiles of four groups at the genus level. The abundance of different subgingival genera in different group. Blue represents lower abundance, and red represents higher abundance.

**Figure 2:**
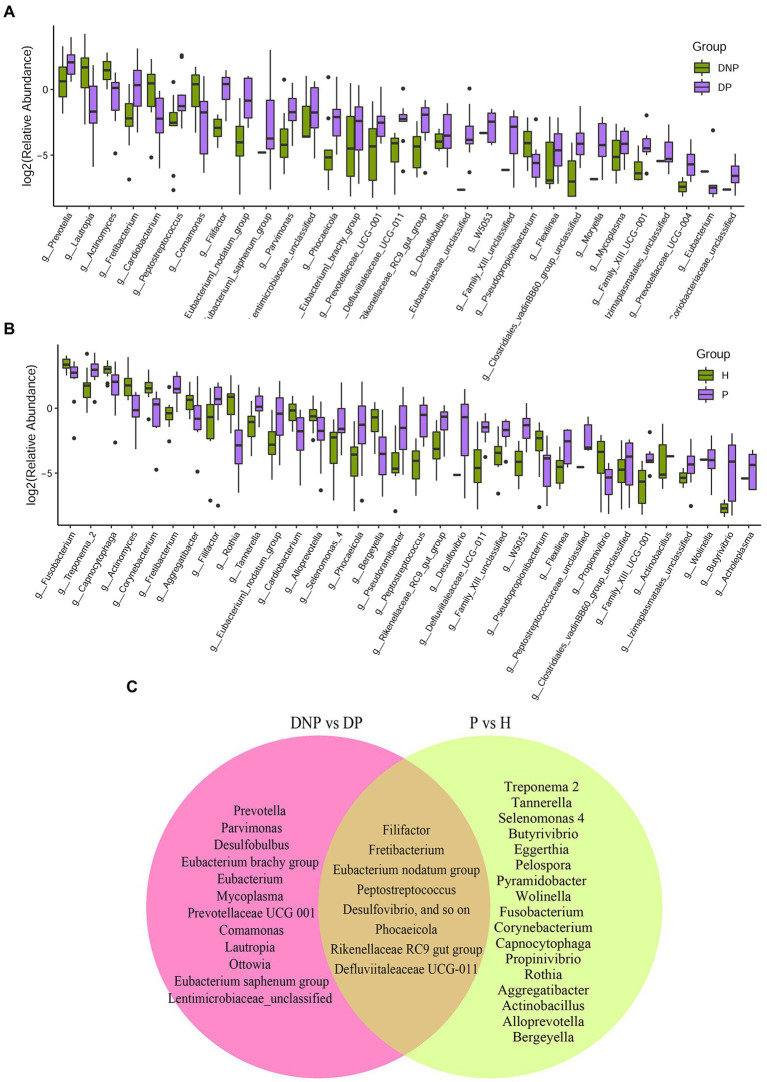
Distribution of differential genera between patients with/without T2DM. **(A)** Difference of relative abundances of subgingival bacterial genera between patients with DP or DNP; **(B)** Difference of relative abundances of subgingival bacterial genera between patients with P or H; **(C)** The 39 genera differentially abundant between the DP and DNP groups; 41 differentially abundant between the P and H groups. There were 23 genera which were both differential genus between the DP and DNP group, and between the P and H groups. Besides, 16 genera were differential genus between the DP and DNP group only; in addition, 18 genera were only differential genus between the P and H groups only.

There were differences in subgingival microbiome composition among the four groups (Figure 1). Altogether, 39 genera were found to be significantly differentially abundant between the DP and DNP group; and 33 genera were more abundant in the DP group, including *Prevotella, Fretibacterium*, *Peptostreptococcus, Filifactor*, *E. saphenum*, *E. nodatum*, and others (Figure 2A). Furthermore, 41 genera were found to be significantly differentially abundant between the P and H groups; and 29 genera were more abundant in the P group, including *Treponema 2*, *Fretibacterium*, *Filifactor*, *E. nodatum*, *Tannerella*, *Pyramidobacter*, *Phocaeicola*, *Pseudoramibacter*, *Selenomonas 4*, *Peptostreptococcus*, *Desulfovibrio*, and so on (Figure 2B). There were 23 genera which were differential genus between the DP and DNP group and between the P and H groups (Figure 2C).

**Figure 3:**
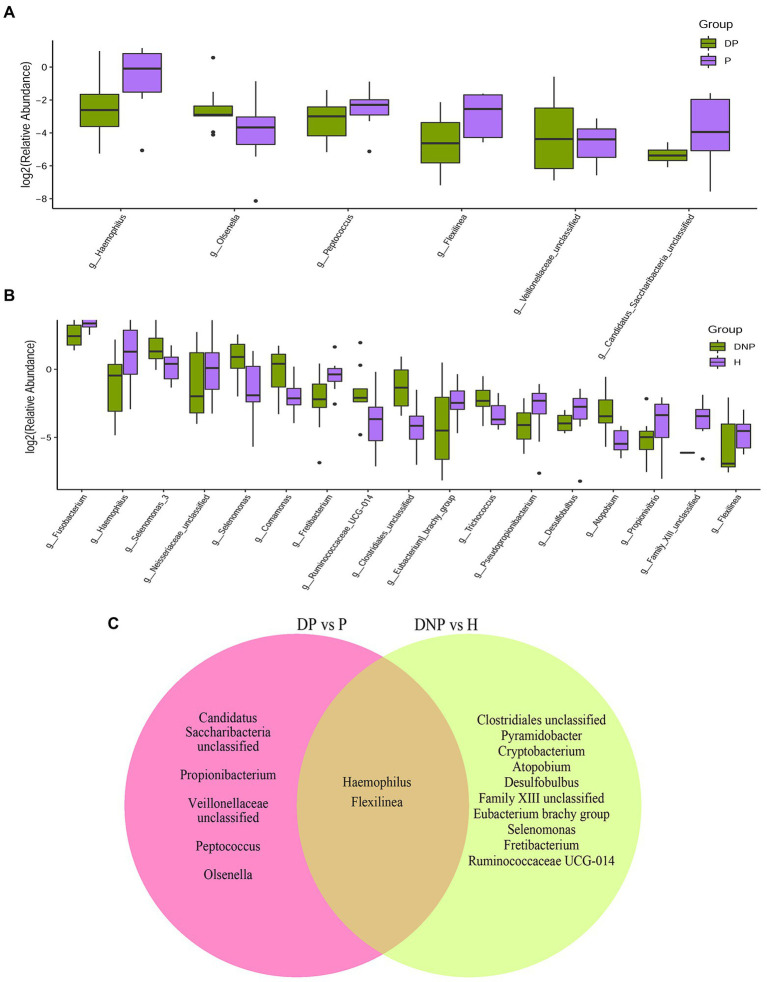
Distribution of differential genera between patients with periodontitis with/without T2DM. **(A)** Difference in relative abundances of subgingival bacterial genera between the DP and P groups; **(B)** Difference in relative abundances of subgingival bacterial genera between the DNP and H groups; **(C)** The nine genera significantly more abundant in the DP vs. P groups; 7 genera significantly more abundant in the DNP than H groups.

We next investigated the microbiome differences between T2DM and ND subjects at the level of bacterial genus. We found 9 genera significantly more abundant in the DP vs. P groups (Figure 3A). Furthermore, 7 genera were significantly more abundant in the DNP vs. H groups (Figure 3B). Lastly, the differential bacteria between T2DM and ND subjects were *Haemophilus* and *Flexilinea* (Figure 3C).

### Microbial analysis of differences between groups

**Figure 4:**
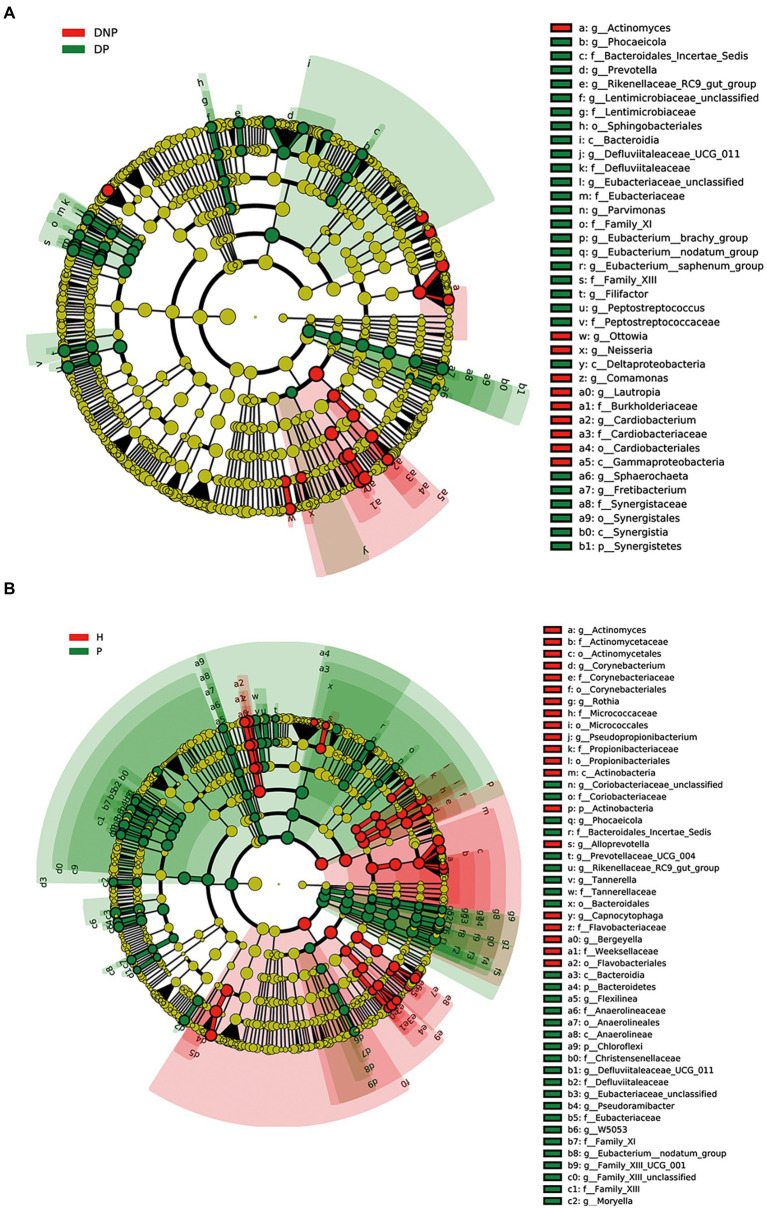
Differences in subgingival micro-organism composition among DP, DNP, P, and H groups. **(A)** Differential subgingival microbial taxa between DP and DNP shown as cladogram by LEfSe analysis; **(B)** Differential subgingival microbial taxa between P and H shown as cladogram by LEfSe analysis. The phylum, class, order, family, and genus levels are listed in order from inside to outside of the cladogram and the labels for levels of class, order, family, and genus are abbreviated by a single letter. The green and red circles represent the bacteria enriched in the group of H or DNP, P or DP, respectively, whereas the yellow circles represent the taxa with no significant differences between groups.

By LEfSe analysis, 20 genera were found to be differentially abundant between the DP and DNP groups. For example, the DP group had a higher abundance of *Prevotella*, *Fretibacterium*, *Peptostreptococcus*, *Filifactor*, *E. saphenum*, *E. nodatum*, and *Phocaeicola*, while a lower abundance of *Lautropia*, *Actinomyces*, *Cardiobacterium*, *Comamonas,* and *Neisseria*. In addition, we found that there were 38 genera differentially abundant between the P and H groups. The P group had a higher abundance of *Treponema 2*, *Fretibacterium*, *Filifactor*, *E. nodatum*, *Tannerella*, *Pyramidobacter*, *Phocaeicola*, *Pseudoramibacter*, *Selenomonas 4*, *Peptostreptococcus*, and *Desulfovibrio*, while a lower abundance of *Fusobacterium*, *Capnocytophaga*, *Actinomyces*, *Corynebacterium*, and *Rothia* (Figure 4).

### Species correlation analysis

**Figure 5:**
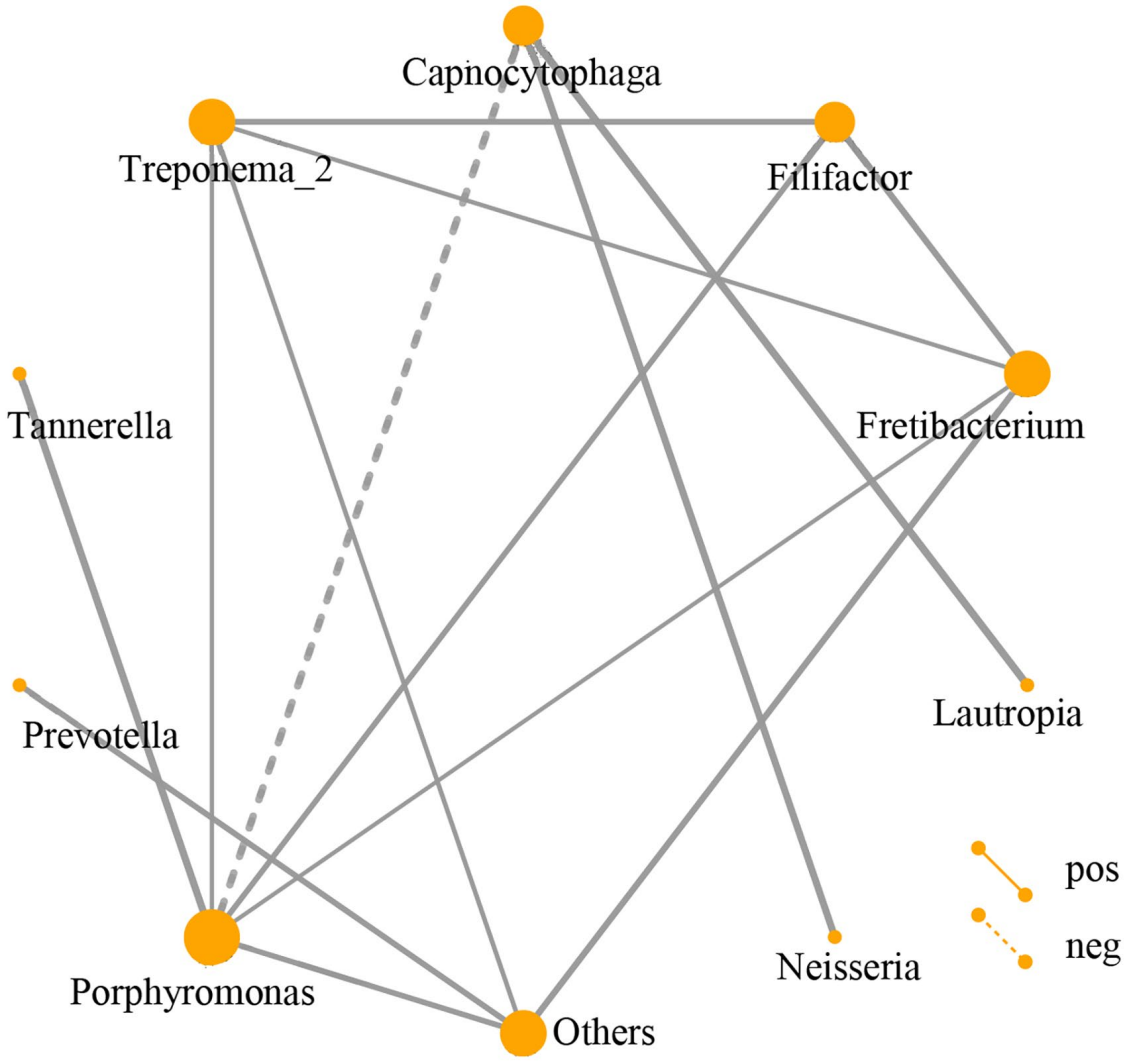
Network analysis of subgingival dominant bacteria genera. Different nodes represent different dominant bacterial genera. The connection between the nodes indicates that there is a correlation between the two genera. By default, we display the relationship pair with a correlation coefficient |rho| > 0.4, and the thickness of the line indicates the strength of the correlation. The thicker the line, the stronger the correlation; the thinner the line, the weaker the correlation. Solid lines indicate positive correlations, dashed lines indicate negative correlations. The size of the node represents the abundance of the flora, the greater the abundance, the larger the node; the lower the abundance, the smaller the node.

The relationship of the top 30 abundant genera among the DP, DNP, P, and H groups by network analysis showed that *Porphyromonas, Tannerella, Filifactor, Fretibacterium, Treponema-2, Capnocytophaga, Lautropia, Neisseria, Prevotella,* and other genera constituted network interaction relationship (Figure 5).

### The gene function prediction of flora

**Figure 6:**
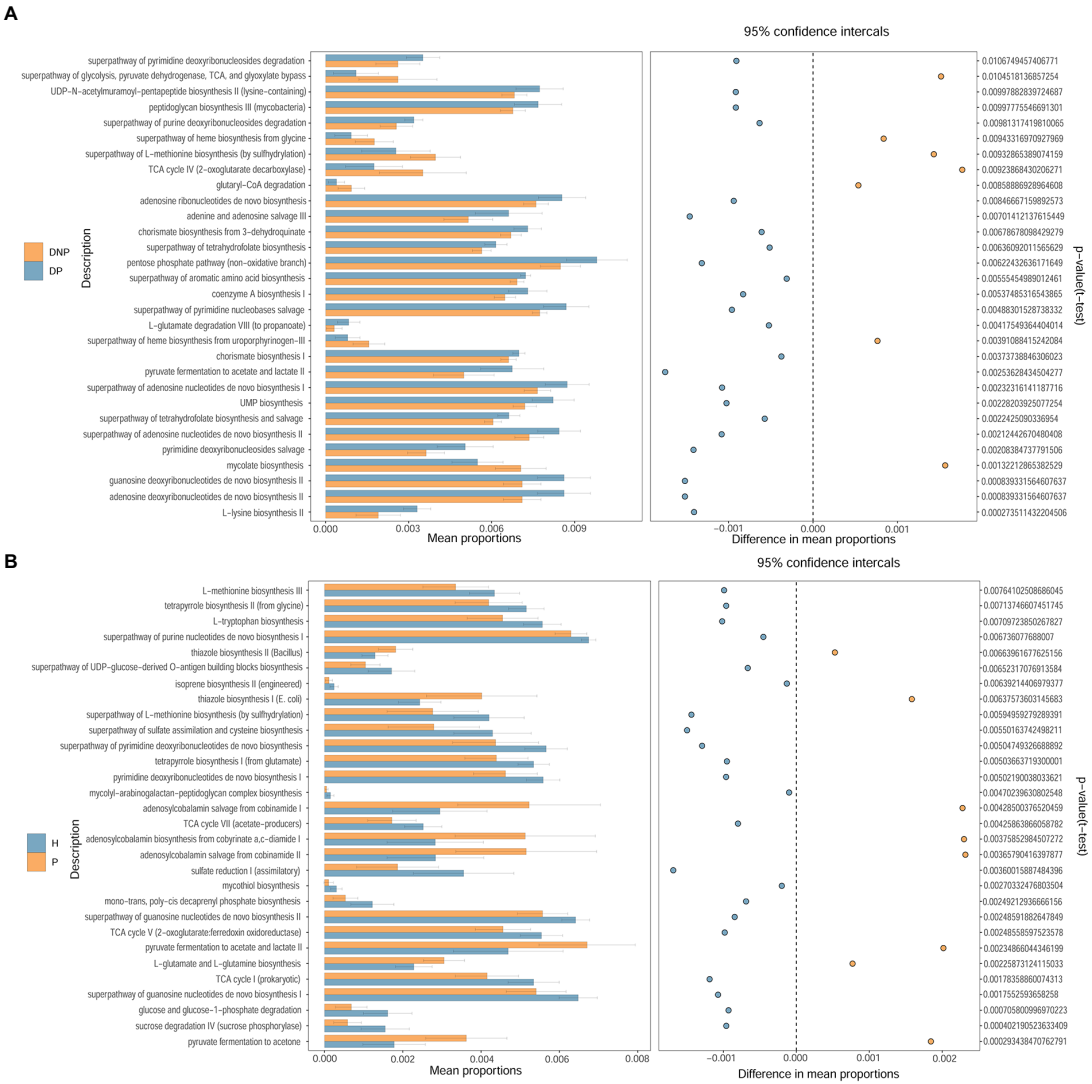
Different pathways between DP and DNP groups, as well as between P and H groups. Comparison of the KEGG Pathway of different bacterial groups and screening out pathways with significant differences between groups. The histogram on the left represents the abundance of metabolic pathways as a percentage of all metabolic pathways in the two sets of samples, while the histogram on the right is the corrected *p*-value. **(A, B)** Comparison of the KEGG Pathway of different bacterial groups and screening out pathways with significant differences between **(A)** DP and DNP; or **(B)** P and H.

To compare the functional changes of the subgingival flora, PICRUSt2 was used to analyze the 16S rRNA gene sequencing data to predict the functions. The results showed that there were significant differences in the function of 30 genes between the DP and DNP groups. The enriched pathways in the DP group included amino acid synthesis and metabolism, as well as guanosine, uridine, pyrimidine deoxyribonucleotide, nucleotide synthesis, pyruvate metabolism, and pentose phosphate metabolism. There were significant differences in the function of 30 genes between the P and H groups. The enriched pathways in the P group included amino acid synthesis and metabolism, glycolysis, nucleotide synthesis, and the tricarboxylic acid cycle (Figure 6).

### Metabolic profiling and Treg/Th17 balance

#### Demographic and clinical characteristics

We investigated the demographic characteristics of all participants, including 10 DP, 9 DNP, 10 P, and 8 H. The FBG and HbA1c of patients with T2DM were higher than those with ND (*p* < 0.05). In addition, there were no significant statistical differences in AL and PD between the DP and P groups (*p* > 0.05; Table 2).

**Table 2:** Demographic and clinical characteristics of subjects.

Group	DP (*n* = 10)	DNP (*n* = 9)	*P* (*n* = 10)	H (*n* = 8)
Age	63.00 ± 9.99	63.67 ± 6.52	49.90 ± 7.50	40.88 ± 6.5
Gender				
Male	6	5	5	4
Female	4	4	5	4
Nationality				
Han	10	8	9	8
Minority	0	1	1	0
FBG (mM)	8.41 ± 2.96	6.47 ± 1.67	5.22 ± 0.26	4.99 ± 0.32
HbA1c (%)	7.01 ± 1.32	6.69 ± 1.02	<6.50	<6.50
PD (mm)	4.04 ± 0.69	2.44 ± 0.05	4.51 ± 0.42	2.19 ± 0.07
AL (mm)	4.27 ± 0.99	2.53 ± 0.22	4.64 ± 0.01	------
BI	2.80 ± 0.35	1.06 ± 0.17	3.10 ± 0.39	0.80 ± 0.23

The FBG and HbA1c of patients with T2DM were higher than ND (*p* < 0.05). In addition, there were no significant statistical differences in AL and PD between the DP and P groups (*p* > 0.05).

### Treg/Th17 balance and The percentage of CD4^+^ /CD8^+^PD1/PDL1

**Figure 7:**
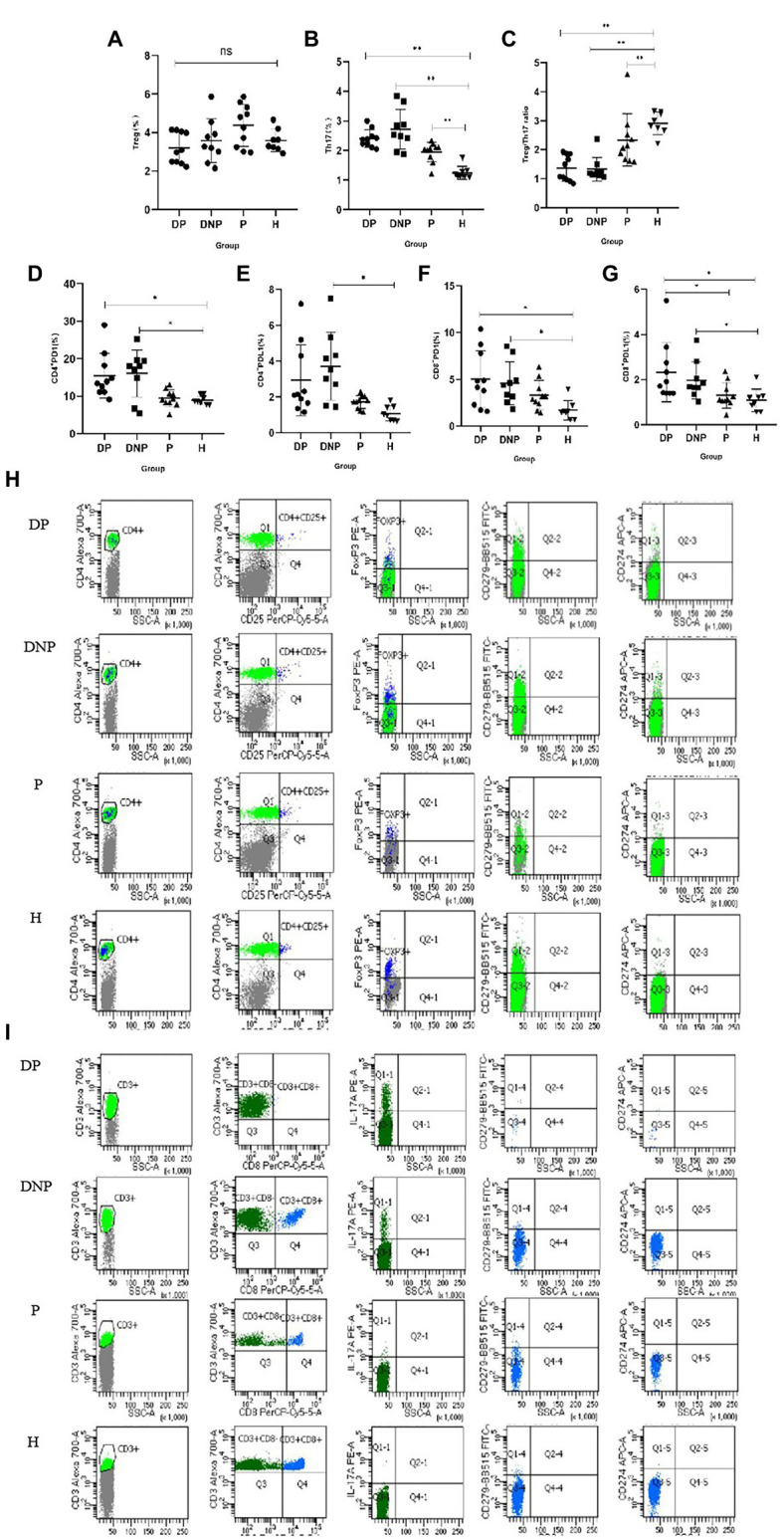
Percentage of Treg and Th17, Treg/Th17 ratio, and expression of PD1 and PDL1 in CD4^+^ and CD8^+^T lymphocytes in peripheral blood. **(A–G)**: The percentage of Treg cells **(A)**, Th17 cells **(B)**, Treg/Th17 ratio **(C)**, and PD1/PDL1 CD4^+^/CD8^+^ **(D–G)** in peripheral blood determined by flow cytometry. **(H)** Comparison of the frequencies of CD4 + CD25 + FoxP3 + T cells, CD4 + PD1/PDL1 cells in the DP, DNP, P, and H. **(I)** Comparison of the frequencies of CD3 + CD8-IL17 + T cells, CD8 + PD1/PDL1 cells in the DP, DNP, P, and H. *p < 0.05, **p < 0.001 (one-way ANOVA test with Tukey’s multiple comparisons test).

The percentage of Th17 in the DP, DNP, and P groups was higher than that in the H group (*p* < 0.001, *p* = 0.001, and *p* < 0.001, respectively). The Treg/Th17 ratio of the DP and DNP groups was lower than that of the P and H groups (*p* < 0.001). The Treg/Th17 ratio of the P group was lower than the H group (*p* = 0.046). The percentage of CD4^+^ PD1 in the DP and DNP groups was higher than in the H group (*p* = 0.042 and *p* = 0.048, respectively), and the percentage of CD4^+^ PDL1 in the DNP group was higher than in the H group (*p* = 0.016). Similarly, the percentage of CD8^+^ PD1 in the DP and DNP groups was higher than in the H group (*p* = 0.03 and *p* = 0.01, respectively); besides, the percentage of CD8^+^ PDL1 in the DP and DNP groups was higher than in the H group (*p* = 0.005 and *p* = 0.044, respectively), and the percentage of CD8^+^ PDL1 in the DP group was higher than the P group (*p* = 0.014; Figure 7).

### Differential analysis of metabolites

**Figure 8:**
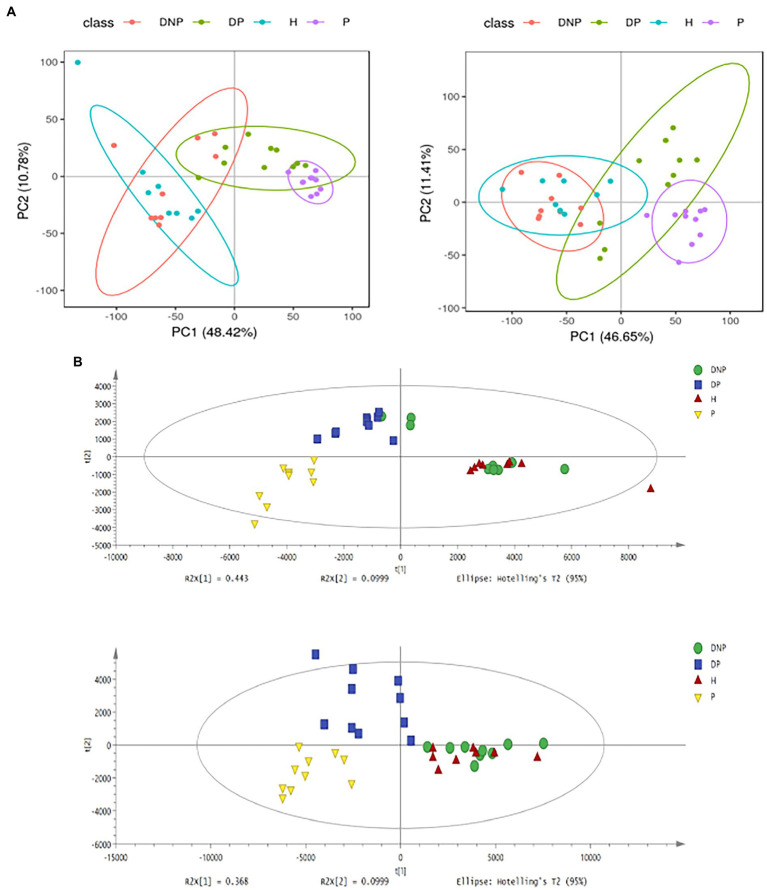
PCA and PLS-DA score plots of plasma samples among groups. **(A)** PCA score plots in ESI+ model (left panel) or ESI– model (right panel); **(B)** PLS-DA shows a significant difference among groups with satisfactory accuracy and prediction (pos R2X: 0.467, R2Y: 0.518, Q2: 0.403; neg R2X: 0.543, R2Y: 0.495, Q2: 0.404).

Multivariate analyses were performed to evaluate the metabolite differences among the four groups. PCA and PLS-DA demonstrated differences between the four groups, respectively. The model presented satisfactory accuracy and prediction (pos. R2X: 0.467, R2Y: 0.518, Q2: 0.403; neg. R2X: 0.543, R2Y: 0.495, Q2: 0.404) (Figures 8A,B).

### Comparison of different metabolites among groups

**Figure 9:**
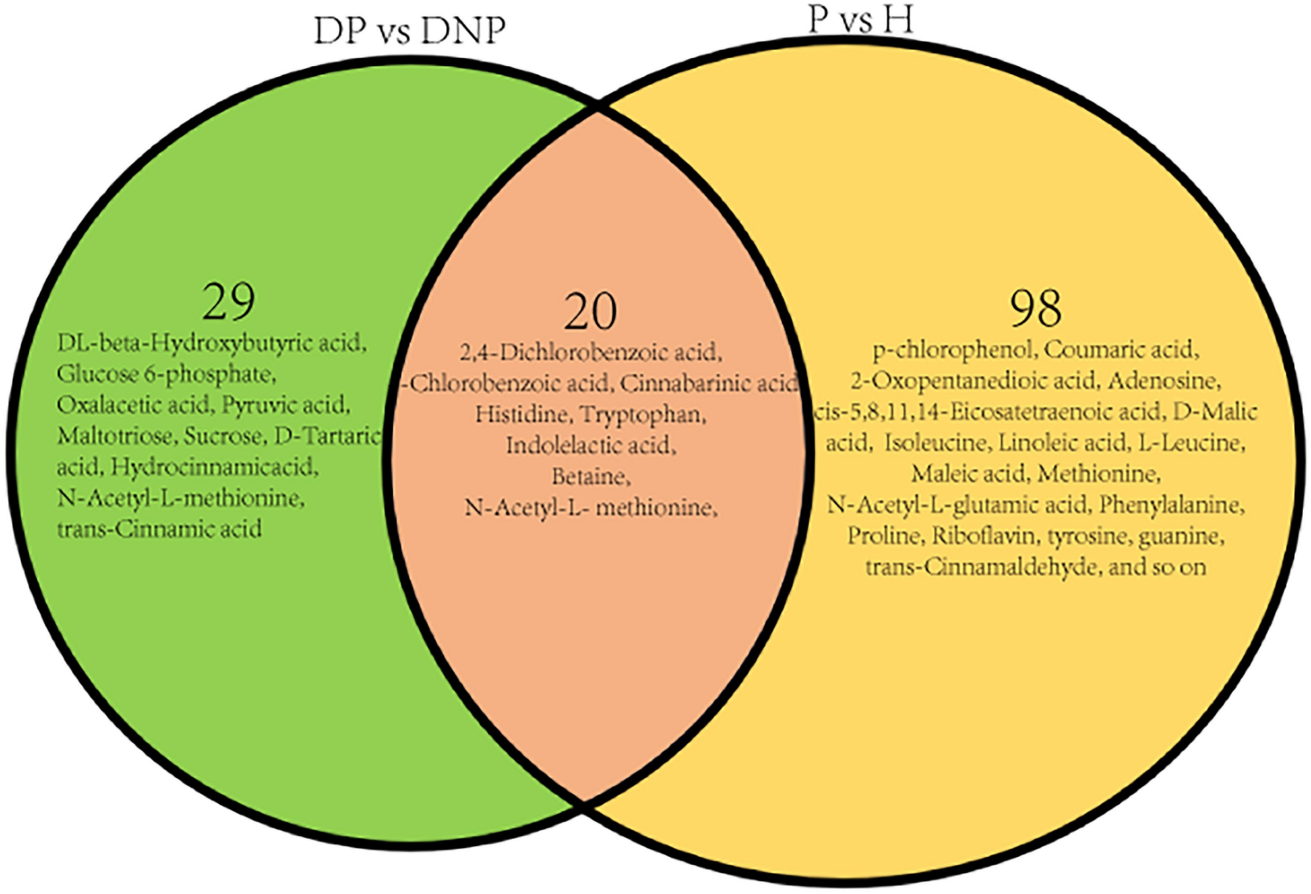
Distribution of differential metabolite in four groups. Distribution of differential metabolite between DP and DNP groups, and between P and H groups. There were 20 metabolite which were both differential metabolite between the DP and DNP group, and between the P and H groups. Besides, 29 metabolite were differential metabolite between the DP and DNP group only; in addition, 98 metabolite were only differential metabolite between the P and H groups only.

The results revealed numerous secondary metabolites between the P and NP groups (Table 3), and the distribution of secondary metabolites between the P and NP groups demonstrated 20 up- or downregulated metabolites (see Figure 9). The levels of 29 metabolites in the DP group were either higher or lower compared to the DNP group only; besides, the levels of 98 metabolites in the P group were either higher or lower than in the H group only (Figure 9). However, there were few differences in metabolite levels between the DNP and H groups, and no metabolic pathway was enriched with metabolites.

**Table 3:** Distribution of secondary metabolites.

Disease status	Groups compared	Total secondary metabolites	Up-regulated	Down-regulated
P vs. NP	DNP/Dp	49	17	32
	P/H	118	84	33
D vs. ND	DNP/H	2	0	2
	DP/P	87	13	74

### KEGG metabolic pathway analysis

**Figure 10:**
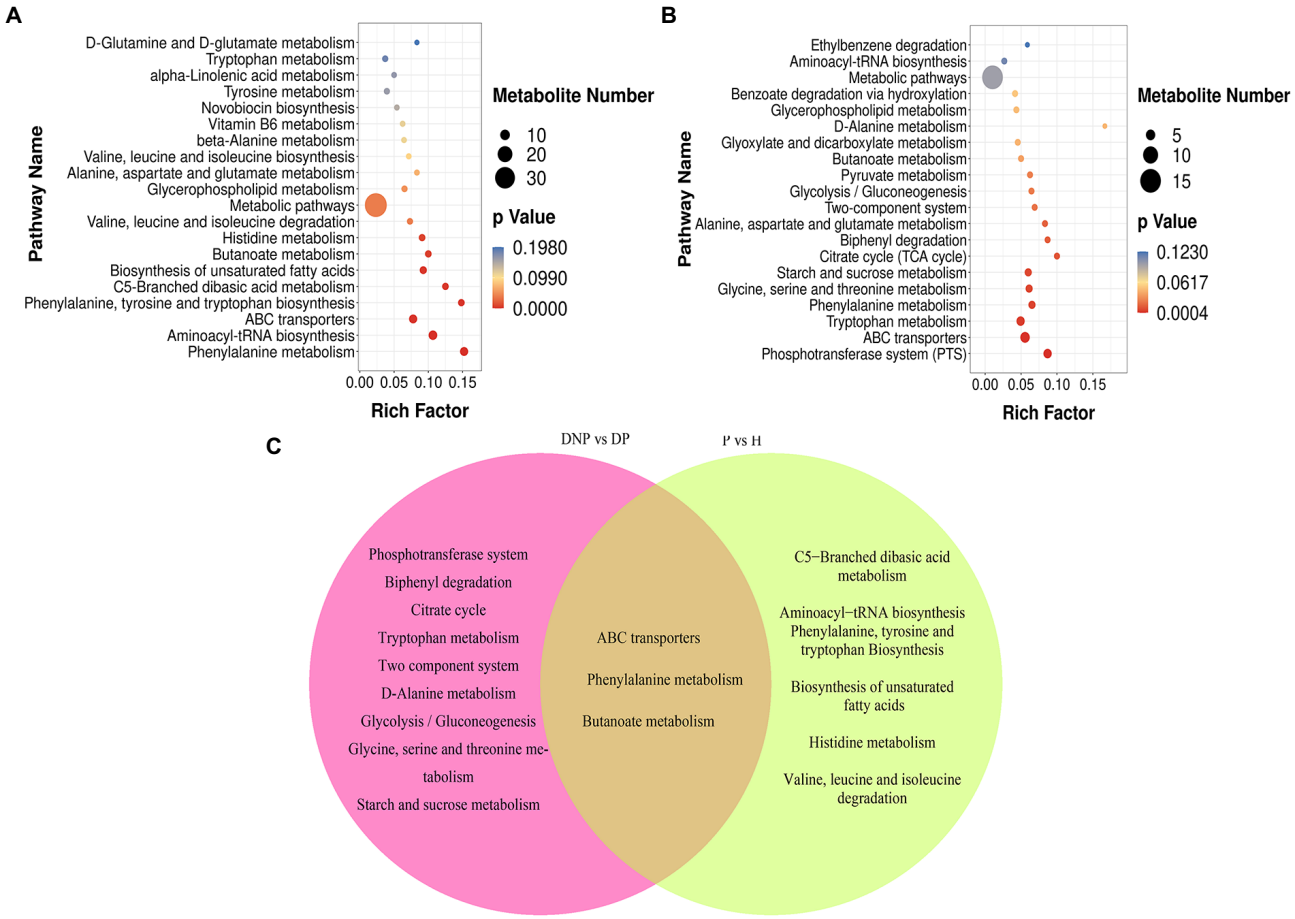
KEGG pathway enrichment of differential metabolites (scatter plot). **(A–C)** KEGG pathway enrichment of differential metabolites including between DNP and DP groups, P and H groups. **(C)** Left part represents KEGG pathway which were both differential pathway between the DP and DNP group, and between the P and H groups. Besides, intersections represent KEGG pathway were differential pathway between the DP and DNP group only; in addition, Right part represents KEGG pathway were only differential pathway between the P and H groups only. The results of KEGG enrichment analysis using ggplot2 are shown in a scatter map. Rich factor = S gene number/B gene number. The left name of the pathway, the smaller the *p*-value, the higher the KEGG enrichment.

KEGG and HMDB pathway analyses were used to analyze related metabolites, and the results were submitted to MetaboAnalyst to display the results of the informatics analysis. The pathway analysis results are shown in Figures 10A–C. The most influenced metabolic pathway was considered a pathway influence cut-off value >0.1 to filter out less important pathways. A total of 16 prominent metabolic pathways were identified between the DP and DNP groups, including the phosphotransferase system, biphenyl degradation, citrate cycle, tryptophan metabolism, two-component system, etc (Figure 10). Furthermore, 10 important metabolic pathways were identified between the P and H groups, including the C5-branched dibasic acid metabolism, aminoacyl−tRNA biosynthesis, phenylalanine, tyrosine, and tryptophan biosynthesis, etc (Figure 10C). There were three metabolic pathways identified between all four groups, including the ABC transport systems, phenylalanine metabolism, and butyrate metabolism (Figure 10C).

### Analysis correlation of different metabolites and clinical indicators

**Figure 11:**
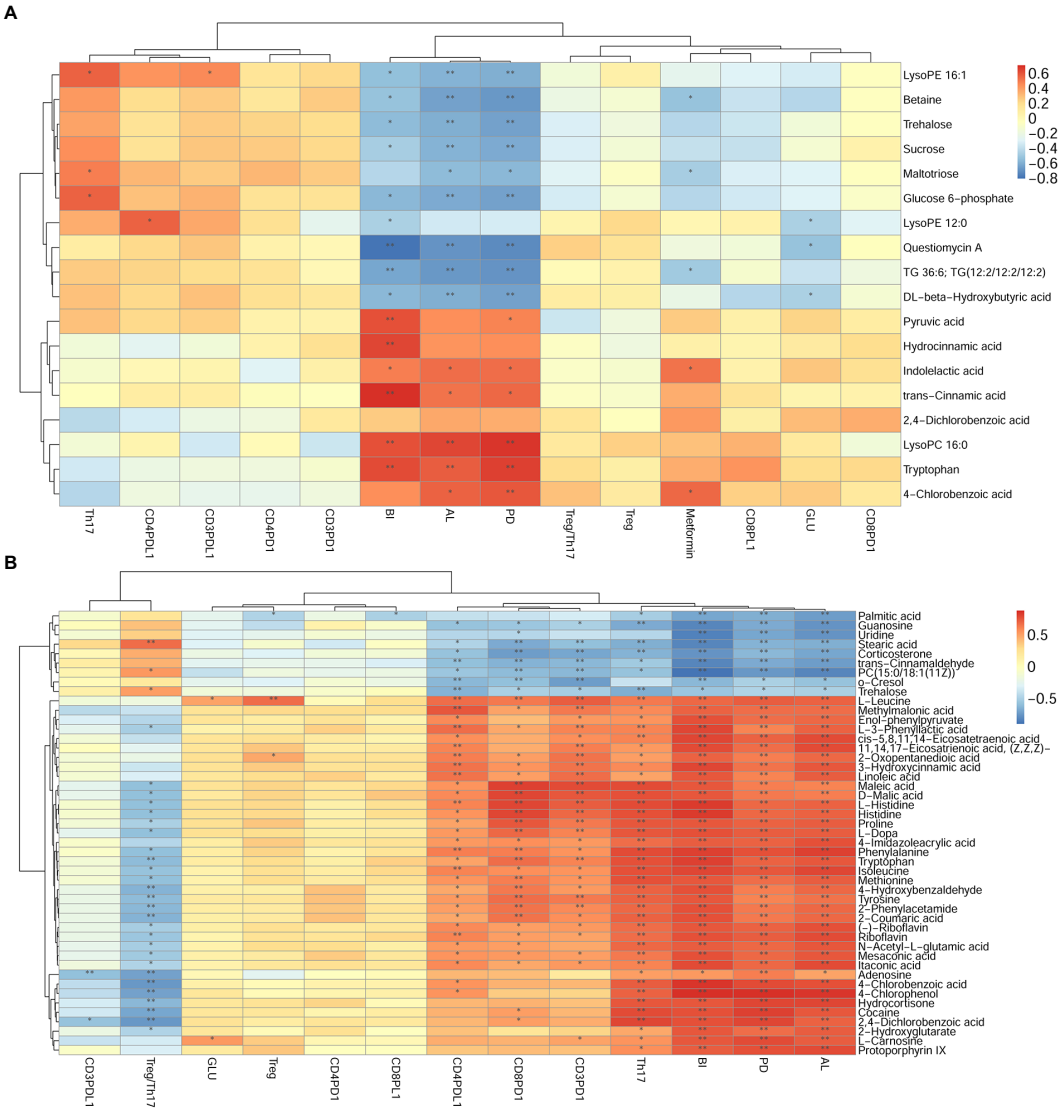
Correlation analysis between differential metabolites and clinical index in DP and DNP, P and H, or DP and P groups. **(A–B)**: Correlation of differential metabolites, clinical index (BI, PD, AL), Th17%, Treg%, Treg/Th17 ratio, and CD4^+^/CD8^+^ PD1/PDL1 expression between **(A)**, DP and DNP, or **(B)**, P and H groups. Blue is negative correlation, red is positive correlation (*p < 0.05, **p < 0.001).

The correlation was determined among metabolites, periodontal clinical treatment, Th17%, Treg%, Treg/Th17 ratio, CD4^+^ PD1%, CD4^+^ PDL1%, CD8^+^ PD1%, CD8^+^ PDL1% by correlation analysis (Figure 11).

### Combined analysis of different bacteria, different metabolites, periodontal clinical treatment, Treg/Th17 ratio, and blood glucose

**Figure 12:**
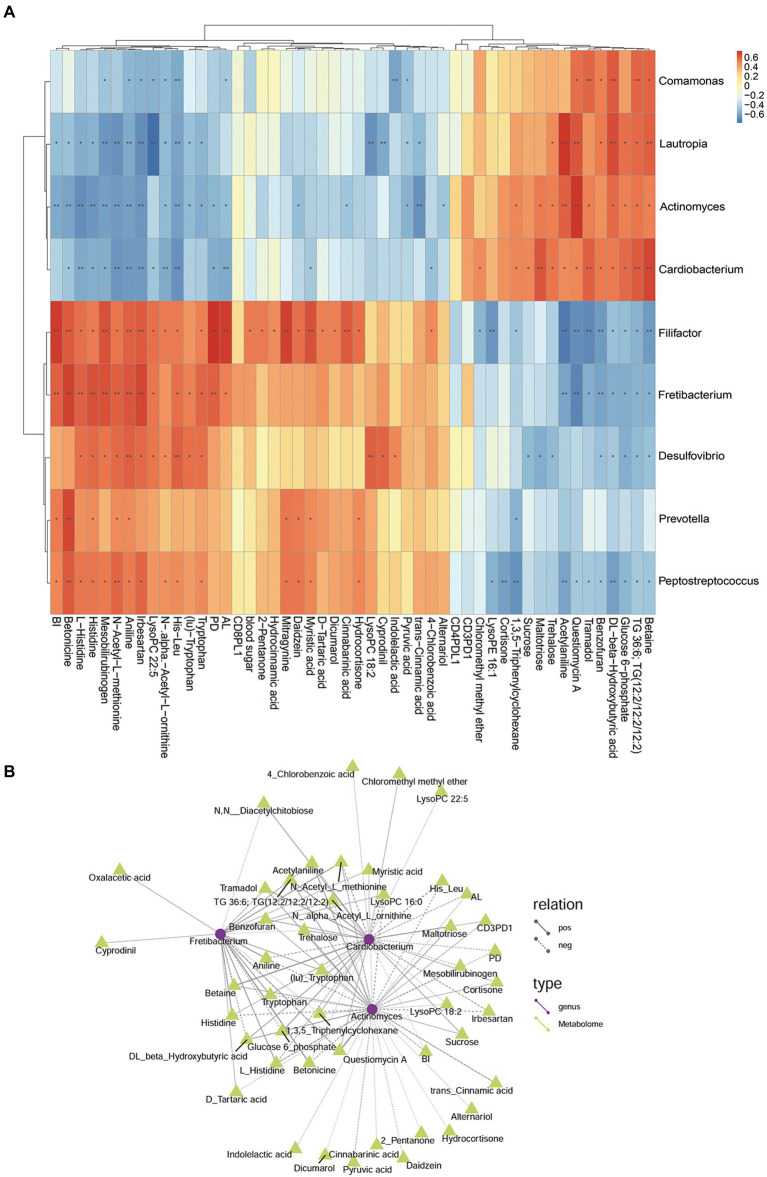
The relationship among genus, metabolites, and clinical index of DP and DNP groups. **(A)** Correlation between differential bacteria and metabolites, with clinical indicators. Red indicates positive correlation, blue indicates negative correlation (*p < 0.05, **p < 0.001). **(B)** Network relationship among differential genera, metabolites, and clinical index of DP and DNP groups. The connection between the microbiota and metabolites represents a correlation between them; the solid line represents a positive correlation, and the dotted line represents a negative correlation.

In the comparison analysis of DP and DNP group, the genus Fretibacterium was negatively related to Actinomyces and Cardiobacterium; meanwhile, Actinomyces was positively related to Cardiobacterium. In addition, Fretibacterium was positively related to AL, BI, PD, Histidine, Tryptophan, D-Tartaric acid, Betonicine, Oxaloacetic acid that involve tryptophan metabolism, Two-component system; however, it was negatively related to Glucose 6_phosphate that involve Phosphotransferase system. Actinomyces and Cardiobacterium was negatively related to AL, BI, PD; they were positively related to Sucrose, Glucose 6-phosphate. Actinomyces was negatively related to Indolelactic acid, trans-Cinnamic acid, Pyruvic acid that involve Butanoate metabolism, phenylalanine metabolism. Cardiobacterium was negatively related to 4-Chlorobenzoic acid that involve Biphenyl degradation (Figure 12).

**Figure 13:**
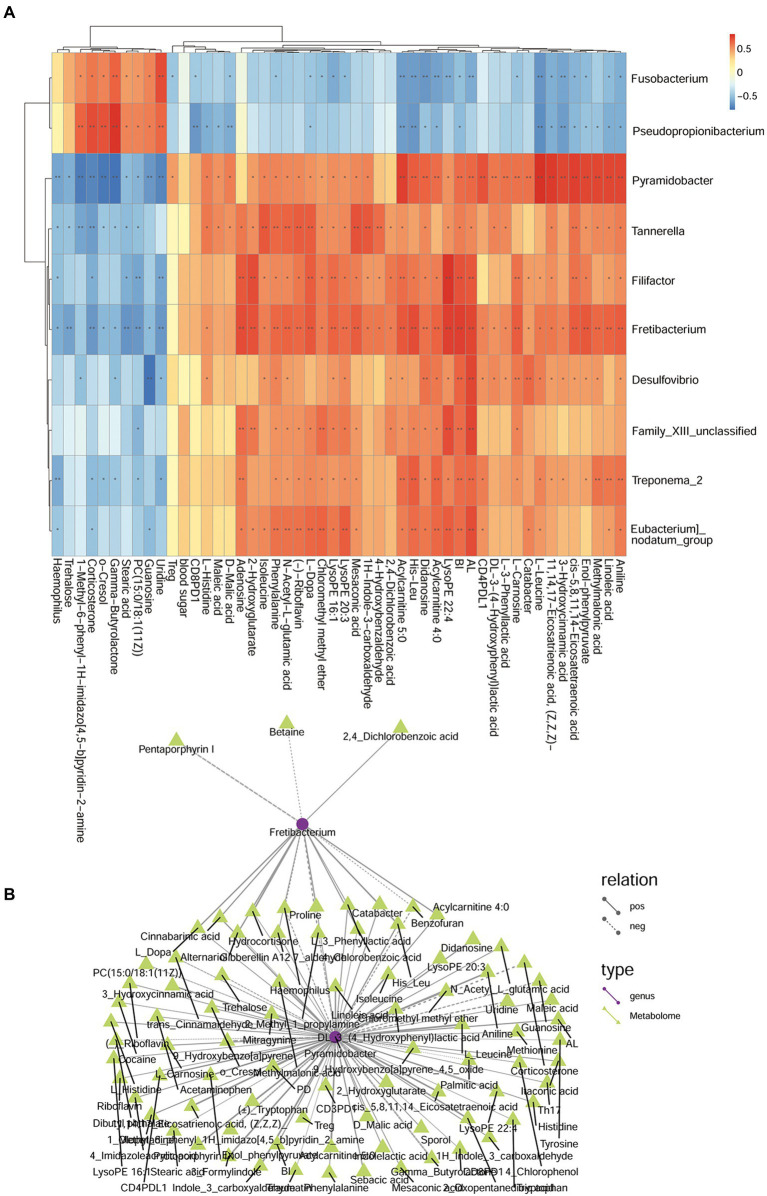
The relationship among genera, metabolites, and clinical index of P and H groups. **(A)** Correlation between differential bacteria and metabolites, with clinical indicators. Red indicates positive correlation, blue indicates negative correlation (*p < 0.05, **p < 0.001). **(B)** Network relationship among differential genera, metabolites, and clinical index of DP and DNP groups. The connection between the microbiota and metabolites represents a correlation between them; the solid line represents a positive correlation, and the dotted line represents a negative correlation.

In the comparison analysis of P and H group, Pyramidobacter was positively related to Fretibacterium, Treg%, Trehalose, Linoleic acid, 3 − Hydroxycinnamic acid, cis − 5,8,11,14 − Eicosatetraenoic acid, 11,14,17 − Eicosatrienoic acid, (Z,Z,Z)−, (−) − Riboflavin, Mesaconic acid, D − Malic acid that involve Biosynthesis of unsaturated fatty acids, Butanoate metabolism, phenylalanine metabolism, Phenylalanine, tyrosine and tryptophan biosynthesis; but it was positively related to O-Cresol, Gamma−Butyrolactone Trehalose. Fretibacterium was positively related to 2,4_Dichlorobenzoic acid (Figure 13). The correlation and network relationship among bacteria, metabolites, periodontal clinical treatment, Th17%, Treg%, Treg/Th17 ratio, CD4^+^ PD1%, CD4^+^ PDL1%, CD8^+^ PD1%, CD8^+^ PDL1%, and blood glucose by correlation and network analysis (Figure 13).

## Discussion

Both diabetes and periodontitis are prevalent chronic diseases affecting epidemic proportions of adults globally (Shi et al., 2020). The two diseases have intertwined pathogenesis: diabetes increasing the risk for periodontitis, periodontal inflammation adversely affecting glycemic control (Lalla and Papapanou, 2011; Shi et al., 2020). While there is a clear relationship between the degree of hyperglycemia and the severity of periodontitis, immune regulation, systemic inflammation, and cytokine biology have also been implicated in the pathogenesis (Taylor et al., 2013; Shi et al., 2020). Nevertheless, the mechanism of the link between the two conditions is unclear.

In this study, we aimed to elucidate the link between T2D and periodontitis from the microbiome perspective. We investigated the subgingival microbiome and metabolome differences between patients with T2D and systemically healthy subjects at the metagenomic and metabolome levels in periodontal health. We found that the subgingival microbiome and metabolome in the periodontitis state differed from that of the healthy state in both groups by varying degrees.

In our study, there were significant differences in the composition of the subgingival microflora between the P and NP groups. However, there were also significant differences in some low-abundance bacteria between the DP and P groups though the compositions and composition ratios were similar. Besides, both patients with DP or P have shown obvious subgingival dysbacteriosis, with subgingival high-abundance of *Porphyromonas*, *Treponema 2*, *Fusobacterium*, *Neisseria*, *Capnocytophaga,* and *Leptotrichia* consistent with the results of other scholars (Boyer et al., 2020). Furthermore, the relative abundance of these genera in the saliva and subgingival plaque of patients with periodontitis was also significantly higher than that of healthy individuals, in accordance with other studies (Socransky et al., 1998; Colombo et al., 2012; Cui et al., 2019). In addition, the abundances of *Desulfovibrio, E. nodatum, Filifactor, Fretibacterium, Peptostreptococcus,* and *Phocaeicola,* predominated in DP and P which may play a key role in periodontitis. *E. brachy, E. saphenum, Parvimonas, Prevotella,* and *Prevotellaceae UCG-001* may also play a significant role in only the occurrence of periodontitis in patients with diabetes. Moreover, *E. nodatum, E. brachy*, and *E. saphenum* are considered novel periodontal pathogens related to periodontitis, which are mainly detected in the subgingival plaque of patients with moderate or severe periodontitis (Hill et al., 1987; Liu et al., 2020). Consistent with the results of other researchers, the abundance of *Porphyromonas, Filifactor, E. nodatum, Tannerella*, and *Treponema* in patients with DP are less than that found in patients with P (Casarin et al., 2013; Park et al., 2015; Graves et al., 2019).

Our results demonstrate that there were positive relationships among *Porphyromonas*, *Tannerella*, *Filifactor*, *Fretibacterium*, and *Treponema 2*, and subgingival micro-organisms were interconnected by parasitic symbiosis, predator symbiosis, harmless symbiosis, competitive symbiosis, etc. (Graves et al., 2019). Therefore, there may be a synergistic or mutually beneficial relationship between these pathogenic bacteria, whereas there may also be a competition or predation relationship between pathogenic bacteria and non-pathogenic bacteria (Faust and Raes, 2012; Liu et al., 2020). Moreover, information can be exchanged among bacteria through network relationships, playing a vital role and affecting the homeostasis of the flora, such as *Porphyromonas* and *Capnocytophaga*. For example, *Porphyromonas gingivalis* is a major pathogen responsible for severe and chronic manifestations of periodontal disease.

In our study, the percentage of Th17 in the DP, DNP, and P groups was higher than in the H group. The Treg/Th17 ratio of the DP and DNP groups was lower than in the P and H groups. The Treg/Th17 ratio of the P group was also lower than the H group. It is known that people with inherent Th17 defects have less periodontal disease (Dutzan et al., 2018), consistent with the importance of Th17 in periodontal inflammation and bone loss (Nikolajczyk and Dawson, 2019). Th17-associated mechanisms heighten the risk of PD in people with T2D. The percentage of CD4^+^ PD1 and CD8^+^ PD1/PDL1 in the DP and DNP group was higher than in the H group, and the percentage of CD8^+^ PDL1 in the DP group was higher than in the P group. PD1 inhibitory signals play critical roles in regulating the threshold for T cell activation and limiting effector T cell responses, as well as controlling T cell tolerance, resolution of inflammation, and T cell exhaustion (Tan et al., 2021). Therefore, PD1 may play a prominent role in T cell immunity of T2DM.

Pathway function prediction revealed that there were significant differences in subgingival microbial metabolism pathway functions between groups, suggesting that periodontitis may be related to aberrant subgingival microbe metabolism. For instance, butyrate metabolism was different between the periodontitis and non-periodontitis groups, as well as between the DP and P groups. Butyrate is an important short-chain fatty acid that can act on a variety of cells, such as gingival fibroblasts, gingival epithelial cells, and T cells. It can induce periodontitis by inhibiting the cell cycle, promoting cell death, stimulating oxidative stress, inducing inflammatory reactions, and destroying periodontal tissue (Tsuda et al., 2010; Shirasugi et al., 2018). In our study, patients with DP or DNP showed correlations between butyrate metabolism-related metabolites and periodontal clinical index (BI, PD, AL), Th17%, Treg/Th17 ratio, CD4^+^ PDL1%, and CD8^+^ PD1%, indicative of the relationship between the severity and immunological state of periodontitis.

Carbohydrate-, glycol-, amino acid-, and energy metabolisms may play important roles in maintaining the balance of microflora. Our results showed abnormalities in the ABC transport system and phenylalanine metabolism between patients with or without periodontitis. In addition, amino acids are not only the basic unit of protein synthesis in the body but also the precursors of a variety of biologically active molecules and metabolic energy substrates. The transcriptome analysis of subgingival plaque shows that amino acid metabolism is closely related to microbial flora imbalance (Szafranski et al., 2015; Sakanaka et al., 2017), and non-target metabolites analysis of saliva from patients with aggressive periodontitis revealed abnormal phenylalanine metabolism (Sakanaka et al., 2017). Besides, subgingival plaque micro-organisms in different disease states have shown amino acid metabolism abnormalities, such as tryptophan, histidine, and phenylalanine metabolism, tryptophan biosynthesis. Our results also showed differences in subgingival microbial PTS metabolism between the DP and DNP groups, which may affect the balance of the flora to a certain extent. The phosphotransferase system (PTS) is a translocation molecule in the carrier family. It plays a role in bacterial transport of carbohydrates, inhibition of catabolism, reserve storage of carbon, as well as coordinating the carbon and nitrogen metabolism balance. Moreover, we found differences in the tricarboxylic acid cycle (TCA) and glycolysis/synthesis between the DP and DNP groups, which was consistent with the results of the saliva metabolome detection of patients with periodontitis (Sakanaka et al., 2017). The TCA is crucial to energy metabolism. It can also bring about a series of chemical reactions to synthesize glucose, fatty acids, and amino acids to meet the needs of cells (Baldwin and Krebs, 1981). Therefore, our study showed evidence of changes in carbohydrate metabolism, amino acid metabolism, energy utilization, and glycometabolism of subgingival plaque micro-organisms in patients with periodontitis.

Biphenyls and polychlorinated biphenyls (PCBs) are common environmental compounds (Xing et al., 2020) with bio-accumulative effects and potential carcinogenicity (Jacobson and Jacobson, 1996; Akahane et al., 2018; Weber et al., 2018). In addition, biphenyls and PCBs can also inhibit the growth of bacterial cells and up-regulate inflammatory genes (Hoffman et al., 2020; Stiborova et al., 2020). For example, PCB153 can cause intestinal epithelial cells to produce an inflammatory response, with additional side-effects on intestinal microbes (Phillips et al., 2018). PCBs can also inhibit the growth of *Serratia*, *Pseudomonas*, and a few *Bacillus* bacteria (Bourquin and Cassidy, 1975; Blakemore and Carey, 1978). Our research showed that 4-chlorobenzoic acid was up-regulated in patients with DP compared to those with DNP, and could serve as positive periodontal clinical indicators. Therefore, subgingival microbial biphenyl metabolism in patients with DP may have a certain effect on the occurrence and development of periodontitis. In addition, the two-component regulatory system (TCS) was significantly different between patients with DP and those with DNP. The TCS is an important signal transduction mechanism widely used by bacteria to maintain their survival in stressful environments. The transmission of chemical signals in response to environmental stress can regulate the transcription of stress-related genes throughout the body (Zschiedrich et al., 2016). Bacteria not only regulate the pathogenic process, virulence, and drug resistance but also regulate the expression of genes related to bacterial amino acid metabolism by the TCS (Lee et al., 2005; Dalebroux and Miller, 2014; Deka et al., 2017). Therefore, the TCS may promote pathogenic effects by regulating the local environment and its virulence (Cotter and Hill, 2003; Li et al., 2015), which play a certain role in the occurrence of periodontitis in patients with T2DM.

The multi-pathway exchange and co-metabolism of compounds between the host and microbiota produces a secondary metabolism that is biologically active in the host and micro-organisms affecting gene regulation. The micro-organisms interact with the host through their metabolic activities, affecting normal physiological activities and disease susceptibility (Lozupone et al., 2012). Metabolites are the bridge between micro-organisms and the host, and there is a two-way relationship between micro-organisms and metabolites. Further network analysis revealed a relationship between the different groups of bacteria and their metabolites, periodontal clinical indicators, Th17%, Treg%, Treg/Th17 ratio, and PD1/PDL1 expression on CD4^+^/CD8^+^T lymphocytes. Furthermore, there was a network relationship among subgingival flora, metabolites, periodontal clinical index, blood glucose level, and T lymphocyte immunity. *Fretibacterium* and *Filifactor* were positively correlated with periodontal clinical index in patients with periodontitis and non-periodontitis, which is consistent with previous studies (Khemwong et al., 2019; Boyer et al., 2020).

In summary, T2DM patients with periodontitis have less obvious subgingival flora imbalance than patients with simple periodontitis. The bacteria E. nodatum, Filifactor, Fretibacterium, Peptostreptococcus, and Desulfovibrio, amongst others, may be of importance in the occurrence and development of periodontitis; the genus of DP subgingival was significantly different from that of DNP. A network relationship occurred among some dominant bacteria. Patients with periodontitis with/without T2DM have obvious metabolic disorders in subgingival microbes, but their marker metabolites are different. Butyrate metabolism, phenylalanine metabolism, and ABC transport system may play a prominent role in periodontitis in T2DM or ND state. Biphenyl degradation, tryptophan metabolism, and the TCS may play a supporting role in T2DM with periodontitis. The subgingival flora, metabolites, blood glucose level, and T lymphocyte immunity presented with an unbalanced network relationship in patients with T2DM and periodontitis.

## Conclusion

In this study, 16SrRNA gene sequencing combined with UHPLC–MS-based metabolomics was used to investigate the subgingival microbiome of patients with T2DM and periodontitis. We found that the change of the subgingival microbiome from healthy status to periodontitis status was less prominent in T2DM compared with ND, and the clinical signs of the disease were similar. *E. nodatum*, *Filifactor*, *Fretibacterium*, *Peptostreptococcus*, and *Desulfovibrio*, amongst others, may play an important role in the pathopoiesia of periodontitis in T2DM. In addition, some dominant bacteria have network relationships. There was an imbalance of Treg/Th17 in T2DM. Subgingival micro-organisms in patients with periodontitis had a significant metabolic shift. The butyrate metabolism and phenylalanine metabolism may play a role in periodontitis with/without T2DM. The biphenyl degradation, tryptophan metabolism, and the TCS may play a key role in T2DM with periodontitis. The network relationship among subgingival micro-organisms, metabolites, blood glucose control, and T lymphocyte immunity were unbalanced.

## Data availability statement

The data present in the study are deposited in the National Library of Medicine repository, accession number: PRJNA889010.

## Ethics statement

The studies involving human participants were reviewed, approved, and conducted by the Ethics Committee of Guangxi Medical University (No. 2020010). The patients/participants provided their written informed consent to participate in this study.

## Author contributions

LJ, JZ, and RT conceived and designed the experiments. LJ and JZ wrote the manuscript. JZ, MF, and YH performed the 16S rRNA gene sequencing experiments. LJ, MF, and YH performed the metabolomics experiments. LJ performed the T lymphocyte immunity experiments. LJ, RT, and YQ analyzed the data. All authors contributed to the article and approved the submitted version.

## Funding

This work was financially supported by the National Science Foundation of China (82160180), Guangxi Medical High-level Talents Training Program, Guangxi Key Laboratory of the Rehabilitation and Reconstruction for Oral and Maxillofacial Research Open Project (GXKLRROM2104), the Basic Ability Improvement Project for Young and middle-aged Teachers in Guangxi Universities (2018KY0106), Guangxi 19th batch of “Ten Thousand Talents Project” special funds, Guangxi Medical High-level Talents Training Program.

## Conflict of interest

The authors declare that the research was conducted in the absence of any commercial or financial relationships that could be construed as a potential conflict of interest.

## Publisher’s note

All claims expressed in this article are solely those of the authors and do not necessarily represent those of their affiliated organizations, or those of the publisher, the editors and the reviewers. Any product that may be evaluated in this article, or claim that may be made by its manufacturer, is not guaranteed or endorsed by the publisher.

## Abbreviations

BMI: body mass index
EDTA: ethylenediaminetetraacetic acid
FBG: fasting blood glucose
HbA1c: glycated hemoglobin
mAb: monoclonal antibody
MS: mass spectrometry
OPLS-DA: partial least squares-discriminate analysis
PBMC: peripheral blood mononuclear cell
PCA: principal component analysis
PCBs: polychlorinated biphenyls
PD1: programmed cell death protein 1
PDL1: programmed cell death ligand 1
PTS: phosphotransferase system
SEM: standard error of the mean
T2DM: type 2 diabetes mellitus
TCA: tricarboxylic acid cycle
TCS: two-component regulatory system
UPLC: ultra-performance liquid chromatography
VIP: variable importance plot
