# Supplementary material for: Analysis of subgingival micro-organisms based on multi-omics and Treg/Th17 balance in type 2 diabetes with/without periodontitis
Source: Front Microbiol. 2022 Nov 28;13:939608. doi: 10.3389/fmicb.2022.939608 (PMC9743466; doi:10.3389/fmicb.2022.939608)
Supplement: Supplementary file 1 [file Data_Sheet_1.PDF]

Table 1 Demographic and clinical characteristics of subjects.

Group	T2DM with periodontitis (DP) (*n*=10)	T2DM without periodontitis (DNP) (*n*=10)	Periodontitis (P) (*n*=10)	Healthy (H) (*n*=11)
Age	63.00±9.99	64.10±6.29	49.9±7.50	44.18±8.14
Gender				
Male	5	5	5	6
Female	5	5	5	5
Nationality				
Han	9	9	9	9
Minority	1	1	1	2
BMI	23.64±2.57	23.63±6.02	22.78±3.01	22.60±3.85
FBG (mM)	8.41±2.96	6.22±1.76	5.22±0.26	5.02±0.29
HbA1c (%)	7.01±1.32	6.69±0.32	<6.50	<6.50
PD (mm)	4.04±0.69	2.42±0.78	4.51±0.42	------
AL (mm)	4.27±0.99	2.51±0.22	4.64±0.61	------
BI	2.80±0.35	0.95±0.16	3.10±0.39	0.82±0.25

Data are presented as mean ± S.E.M. unless otherwise indicated.

BI, bleeding index; PD, probe depth of the sampling site; AL, attachment loss; BMI, body mass index; T2DM, type 2 diabetes mellitus.

The age of patients with T2DM was higher than those without diabetes ($P<0.05$); The FBG and HbA1c levels were higher in patients with DP than DNP ($P<0.05$); There was no significant difference in PD, AL, or BI between the DP and P groups ($P>0.05$).
