# Supplementary material for: Analysis of subgingival micro-organisms based on multi-omics and Treg/Th17 balance in type 2 diabetes with/without periodontitis
Source: Front Microbiol. 2022 Nov 28;13:939608. doi: 10.3389/fmicb.2022.939608 (PMC9743466; doi:10.3389/fmicb.2022.939608)
Supplement: Supplementary file 2 [file Data_Sheet_2.PDF]

Table 2 Demographic and clinical characteristics of subjects.

Group	DP (*n*=10)	DNP (*n*=9)	P (*n*=10)	H (*n*=8)
Age	63.00±9.99	63.67±6.52	49.90±7.50	40.88±6.56
Gender				
Male	6	5	5	4
Female	4	4	5	4
Nationality				
Han	10	8	9	8
Minority	0	1	1	0
FBG (mM)	8.41±2.96	6.47±1.67	5.22±0.26	4.99±0.32
HbA1c (%).	7.01±1.32	6.69±1.02	<6.50	<6.50
PD (mm)	4.04±0.69	2.44±0.05	4.51±0.42	2.19±0.07
AL (mm)	4.27±0.99	2.53±0.22	4.64±0.01	------
BI	2.80±0.35	1.06±0.17	3.10±0.39	0.80±0.23

The FBG and HbA1c of patients with T2DM were higher than ND ($P<0.05$). In addition, there were no significant statistical differences in AL and PD between the DP and P groups ($P>0.05$).
