# Supplementary material for: Analysis of subgingival micro-organisms based on multi-omics and Treg/Th17 balance in type 2 diabetes with/without periodontitis
Source: Front Microbiol. 2022 Nov 28;13:939608. doi: 10.3389/fmicb.2022.939608 (PMC9743466; doi:10.3389/fmicb.2022.939608)
Supplement: Supplementary file 3 [file Data_Sheet_3.PDF]

Table 3 Distribution of secondary metabolites

Disease status	Groups compared	Total secondary metabolites	Up-regulated	Down-regulated
P vs NP	DNP/DP	49	17	32
	P/H	118	84	33
D vs ND	DNP/H	2	0	2
	DP/P	87	13	74
